# Is social inequality related to different patient concerns in routine oral cancer follow-up clinics?

**DOI:** 10.1007/s00405-016-4208-x

**Published:** 2016-07-22

**Authors:** Sarah Allen, Derek Lowe, Rebecca V. Harris, Steve Brown, Simon N. Rogers

**Affiliations:** 1 Department of Health Services Research, Institute of Psychology Health and Society, University of Liverpool, Room 111, 1st floor, Block B, Waterhouse Building, 1-5 Brownlow Street, Liverpool, L69 3GL UK; 2 Faculty of Health and Social Care, Evidence-Based Practice Research Centre (EPRC), Edge Hill University, St Helens Road, Ormskirk, L39 4QP UK; 3 Regional Maxillofacial Unit, University Hospital Aintree, Liverpool, L9 1AE UK; 4 Department of Psychological Sciences, University of Liverpool, Liverpool, L69 3BX UK

**Keywords:** Patient concerns inventory, Social deprivation, Patient reported outcomes, Health-related quality of life, Oral cancer

## Abstract

Oral cancer has a higher incidence in the lower social strata, and these patients are less likely to engage in supportive interventions and report a poorer quality of life (QoL). The aim of this paper is to compare the Patient Concerns Inventory (PCI) responses across social groups attending routine oral cancer follow-up clinics with particular focus on the deprivation lower quartile. The PCI package is completed by patients as part of their routine review consultation with SNR. Patients were those diagnosed between 2008 and 2012. Deprivation was stratified using the IMD 2010 from postcode. Of the 106 eligible patients, 85 % used the PCI. Just over half (54 %) were living in the most deprived quartile, with two-thirds (68 %) of males in the most deprived quartile, compared with 35 % of females (*p* = 0.004). In regard to number and type of PCI items selected by patients at their first PCI clinic, there were no notable differences in respect of IMD classification. The two commonest concerns were fear of recurrence (43 %) and sore mouth (43 %). The most deprived quartile reported significant problems in regard to mood (*p* = 0.004) and recreation (*p* = 0.02), and a non-significant trend (36 vs 18 %, *p* = 0.09) in stating their overall QoL as being less than good. It is possible to identify the concerns of patients from lower socioeconomic strata as part of routine follow-up clinics. This allows for targeted multi-professional intervention and supports to improve the outcome in this hard to reach group.

## Introduction

Cancer affecting the head and neck is a condition which seriously impacts a number of areas of life, ranging from physical health to emotional wellbeing, both as a result of cancer and of treatment. This results in the patient experiencing a number of concerns, such as trismus, fear of recurrence, issues with speech and feeding and xerostomia [1]. A recent study found that individuals from low socioeconomic backgrounds were more likely to develop head and neck cancer than those higher up the socioeconomic gradient; this relationship seems to be mediated by differences in smoking and alcohol consumption [2]. Furthermore, head and neck cancer patients from deprived backgrounds experience worse survival rates and lower health-related quality of life (HRQoL) than other patients higher up the socioeconomic gradient [3–5].

Socioeconomic status (SES) and deprivation are concepts which take into account factors, such as an individual’s income, education level, and type of occupation. It describes not only what resources are available to an individual, but also how those resources might limit them [6]. One measure of SES is indices of multiple deprivation (IMD) which is updated by the UK government every few years, and is comprised of income, employment, health, education, housing and services, living environment, and crime [7]. This can be used in conjunction with an individual’s postcode to determine how deprived their area of residence is.

Patients from low socioeconomic backgrounds are less likely to participate in health-related postal surveys [4] and focus groups [8]. This may be due to poorer health literacy and recall of symptoms [9] or low self-esteem [10], which make patients less inclined to express their concerns either in research or to a healthcare professional. If a patient does not express their needs to a healthcare professional, then these needs cannot be addressed, therefore potentially hampering their recovery from cancer. This may partly explain why low SES patients experience worse HRQoL [3].

The patient concerns inventory (PCI) is a tool which was developed for use in clinical appointments to help the patient express any concerns which they might be experiencing, and allows the patient to discuss any issue which they feel is of particular importance. It is a 56-item question prompt list which is completed by the patient in the waiting room before their appointment, and allows the patient to select which concerns they wish to discuss ranging from physical symptoms, social issues, treatment-related concerns, and psychological issues. There is also a section for patients to select any specific healthcare professionals they wish to see. A version of the PCI has been developed specifically for head and neck cancer [11].

Previous studies have found that use of the PCI is feasible with elderly patients and those who have not achieved a high level of education [12, 13], and one study has looked at differences in PCI responses by age group [14]; however, to date, there have not been any studies which have examined how PCI responses might differ by patient’s socioeconomic status. Such a study may contribute to our understanding of whether there are differences in how patients across the socioeconomic gradient use the PCI. Therefore, the present study aims to investigate how PCI responses differ across the socioeconomic gradient in patients attending routine oral cancer follow-up clinics, focusing particularly on patients from the lower end of the gradient.

## Patients and methods

The University Hospital Aintree database was used to access records of patients treated for primary head and neck squamous cell oral carcinoma between 2008 and 2012 with SNR as the responsible consultant. Patients with cutaneous and salivary gland malignancy or living overseas were excluded. The study was approved by the Clinical Audit Department, University Hospital Aintree. Informed consent was obtained from participants.

Patient postcodes at diagnosis were used to obtain 2010 Indices of Deprivation ranks and scores for patients resident in England, such data being publically available (Department for Communities and Local Government). These indices provide a relative overall measure of deprivation (the IMD 2010) at small area level across England. Areas are ranked from least deprived (rank 32,482) to most deprived (rank 1). The IMD 2010 score is constructed from 7 component scores. To facilitate the presentation of results, the IMD 2010 ranks were grouped under national quartiles—the most deprived quartile of local areas ranked 1–8210 in England and the least deprived quartile ranked 24633–32482 in England. Due to the proximity of Wales, some IMD scores were not obtainable.

The PCI is a checklist comprising 56 specified items of patient concern and 18 professionals tiled alphabetically. Previous work (REF) grouped the items of concern into domains: (a) physical and functional well-being (29 items); (b) psychological and emotional well-being/spiritual (14 items); (c) social care/social well-being (9 items); and (d) treatment-related (4 items). The PCI asks respondents to indicate items from the checklist that they were concerned about and wanted to discuss with the doctor during their consultation. Patients were also asked to indicate which professionals from the checklist that they would like to speak with or be referred to. Two items (dry mouth and sore mouth) were added to the PCI in the middle of 2012; otherwise, most items date from 2007, and a few from March 2008.

The University of Washington Quality of Life questionnaire (UW-QOL) version 4 has 12 single question domains, with between 3 and 6 response options scaled evenly from 0 (worst) to 100 (best) [15]. Patients also choose up to three domains of most importance to them in the previous week. Earlier work [16] derived criteria to determine in which domains patients had a ‘significant problem’ or ‘dysfunction’, these criteria being based on a mix of domain scores and domain importance. There is also a single item overall QOL question (very poor, poor, fair, good, very good, outstanding) in which patients were asked to consider not only physical and mental health, but also other factors, such as family, friends, spirituality or personal leisure activities important to their enjoyment of life. UW-QOL data are presented within two subscales, physical function and social–emotional function as derived from earlier work [17] by which a physical function score is obtained by averaging the swallowing, chewing, speech, saliva, taste and appearance domain scores, and a social–emotional function score by averaging the activity, recreation, pain, mood, anxiety, and shoulder domain scores.

Kaplan–Meier methods were used to estimate cumulative survival with survival curves compared using the log-rank test. Fisher’s exact test or the Chi-squared test as appropriate was used to compare patient and clinical characteristics according to use or not of PCI, and also according to IMD deprivation status (most deprived quartile Q1 vs less deprived quartiles Q2–Q4). The Mann–Whitney test was used to compare patient age by use of the PCI, and number of PCI items (overall and for domains) by IMD status. Spearman correlation was used to measure the strength of association between actual overall IMD score and the total number of PCI items selected by patients.

## Results

There were 131 patients in the oral cancer cohort with SNR as the primary consultant at the time of primary diagnosis during 2008–2012. Of these, 15 were palliation cases, 7 others were followed up elsewhere (including IOM), 2 had cognitive impairment, and 1 was described as being an ambulance/SPR stream patient. This left a cohort of 106 patients potentially eligible for PCI, of whom 85 % (90) used the PCI. Table 1 compares the use of PCI in respect of patient and clinical characteristics, and there was a significant difference in regard to age at diagnosis with notably fewer elderly patients over the age of 75 using the PCI and on average a 15-year age difference between PCI users (median 63 years) and non-users (median 78 years). There was also a borderline significance difference in respect of clinical staging with fewer patients with advanced tumours making use of the PCI. Using Kaplan–Meier methods, survival for the 90 patients who used the PCI was estimated as 92 % (SE 3 %) at 12 months, and 84 % (SE 4 %) at 24 months. This was significantly different (*p* = 0.001 log-rank test) from the 16 patients not using the PCI for whom survival was 50 % (SE 13 %) at 12 months, and 44 % (SE 12 %) at 24 months. Specifically of the 16 not using the PCI, 8 died within 12 months (median age at diagnosis 75 range 53–84), 2 died at 18 months (age 93) and 54 months (age 86), and 6 , all with at least 34 months of follow-up, were aged 61, 66, 81, 83, 86, and 91 years at diagnosis.

**Table 1:** Patient and clinical characteristics of the cohort of 106 oral cancer patients for whom the PCI could have been used

	% Using PCI	*p* value
All patients	85 % (90/106)	
Gender		
Male	85 % (50/59)	>0.99*
Female	85 % (40/47)	
Age		
Median (IQR)	PCI: 63 (56–73) *n* = 90	<0.001***
	No PCI: 78 (69–84), *n* = 16	
<55	95 % (18/19)	
55–64	97 % (34/35)	
65–74	80 % (20/25)	
75–84	71 % (15/21)	
85+	50 % (3/6)	
IMD quartile based on national ranks		
Q1 most deprived	89 % (47/53)	0.69**
Q2	87 % (13/15)	
Q3	80 % (16/20)	
Q4 least deprived	79 % (11/14)	
Q1 most deprived	89 % (47/53)	0.41*
Q2–Q4	82 % (40/49)	
IMD not known	75 % (3/4)	
Tumour site		
Buccal	82 % (14/17)	0.89**
Lower gum	85 % (11/13)	
Tongue (ant 2/3)	83 % (40/48)	
FOM	87 % (20/23)	
Other	100 % (5/5)	
Overall TN stage		
1–2	90 % (64/71)	0.08* excluding not known
3–4	76 % (26/34)	
Not known	– (0/1)	
Primary treatment		
Surgery only	86 % (54/63)	0.93**
Surgery + RT/CRT	84 % (32/38)	
RT/CRT not surgery	80 % (4/5)	
Year of operation or diagnosis if no surgery		
2008	87 % (26/30)	0.42**
2009	83 % (19/23)	
2010	85 % (22/26)	
2011	73 % (11/15)	
2012	100 % (12/12)	

* Fisher’s exact test

** Chi squared test

*** Mann–Whitney test comparing age distributions between the two IMD groups

Of the 90 patients using the PCI, 87 had IMD deprivation data derived from postcodes of residence at diagnosis. The rest of this paper focuses on these 87 patients and the relationship between IMD classification and first clinic use of PCI/UW-QOL. The median (IQR) time from surgery (or from diagnosis if no surgery) to first clinic was 4.0 (1.7–9.6) months. At the time of diagnosis, just over half (54 %, 47/87) of patients were living in the most deprived quartile of residential areas based on national ranks for England as a whole, with 15 % (13) in the 2nd quartile, 18 % (16) in the 3rd quartile, and 13 % (11) in the least deprived 4th quartile of areas. Table 2 describes the patient and clinical characteristics of the 87, as well as looking at what characteristics associate with residence in the most deprived quartile. Two-thirds (68 %) of males lived in the most deprived quartile, compared with 35 % of females (*p* = 0.004) and those in the most deprived quartile were 4 years younger on average (*p* = 0.14) with 63 % of those aged under 65 years and 42 % of those aged 65 years and over living in the most deprived quartile.

**Table 2:** Patient and clinical characteristics and IMD 2010 status of the cohort of 87 oral cancer patients who used the PCI and for whom there were IMD deprivation data

	% Living in IMD Q1 most deprived quartile based on National ranks	*p* value
All patients	54 % (47/87)	
Gender		
Male	68 % (34/50)	0.004*
Female	35 % (13/37)	
Age		
Median (IQR)	IMD Q1: 62 (55–69), *n* = 47	0.14***
	IMD Q2–Q4: 66 (58–75), *n* = 40	
<55	65 % (11/17)	
55–64	62 % (21/34)	
65–74	39 % (7/18)	
75+	44 % (8/18)	
Tumour site		
Buccal	62 % (8/13)	0.40**
Lower gum	55 % (6/11)	
Tongue (ant 2/3)	45 % (17/38)	
FOM	70 % (14/20)	
Other	40 % (2/5)	
Overall TN stage		
1–2	49 % (30/61)	0.24* excluding not known
3-4	65 % (17/26)	
Primary treatment		
Surgery only	55 % (28/51)	0.97**
Surgery + RT/CRT	53 % (17/32)	
RT/CRT not surgery	50 % (2/4)	
Year of operation or diagnosis if no surgery		
2008	60 % (15/25)	0.83**
2009	53 % (9/17)	
2010	45 % (10/22)	
2011	64 % (7/11)	
2012	50 % (6/12)	

* Fisher’s exact test

** Chi-squared test

*** Mann–Whitney test comparing age distributions between the two IMD groups

In regard to number of PCI items selected by patients at their first PCI clinic, there were no notable differences in respect of IMD classification (Table 3). Spearman correlation coefficient between IMD score and total number of PCI items was *r*_s_ = −0.01, *p* = 0.93, *n* = 87. The most common concerns raised by patients on the PCI are shown in Table 4, while the members of staff patients most wanted to see or be referred to are shown in Table 5. There was considerable overlap between IMD groups in the items of concern raised by patients and in whom they most wanted to see. There were no statistically significant differences between IMD groups in respect of any specific PCI item (results not shown).

**Table 3:** The number of PCI items selected overall, and for each PCI domain, by IMD 2010 deprivation group

	Most deprived national IMD quartile Q1 (*N* = 47) Median (IQR) IMD score: 54 (43–64)	Less deprived national IMD quartiles Q2–Q4 (*N* = 40) Median (IQR) IMD score: 14 (9–19)	*p* value*
(A) Physical and functional well-being (29 items)			
No items selected	19 % (9)	8 % (3)	
One	21 % (10)	20 % (8)	
Two	13 % (6)	20 % (8)	
Three–four	15 % (7)	23 % (9)	
Five–nine	28 %(13)	28 % (11)	
Ten–twelve	4 % (2)	3 % (1)	
Median (IQR), mean	2 (1–6), 3.51	3 (1–5), 3.50	0.61
(B) Psychological and emotional well-being/spiritual (14 items)			
No items selected	36 % (17)	38 % (15)	
One	40 % (19)	33 % (13)	
Two	6 % (3)	20 % (8)	
Three–six	17 % (8)	10 % (4)	
Median (IQR), mean	1 (0–1), 1.17	1 (0–2), 1.02	0.97
(C) Social care/social well-being (9 items)			
No items selected	66 % (31)	68 % (27)	
One	26 % (12)	30 % (12)	
Two–three	9 % (4)	3 % (1)	
Median (IQR), mean	0 (0–1), 0.47	0 (0–1), 0.35	0.73
(D) Treatment-related (4 items)			
No items selected	87 % (41)	73 % (29)	
One	9 % (4)	23 % (9)	
Two	4 % (2)	5 % (2)	
Median (IQR), mean	0 (0–0), 0.17	0 (0–1), 0.33	0.10
Total number of PCI items (56 items)			
No items selected	9 % (4)	8 % (3)	
One	13 % (6)	–	
Two	21 % (10)	13 % (5)	
Three–four	9 % (4)	30 % (12)	
Five–nine	32 % (15)	35 % (14)	
Ten–nineteen	17 % (8)	15 % (6)	
Median (IQR), mean	4 (2–8), 5.32	4 (3–8), 5.20	0.46
Total number of health professionals			
No items selected	51 % (24)	50 % (20)	
One	30 % (14)	33 % (13)	
Two	11 % (5)	10 % (4)	
Three–five	9 % (4)	8 % (3)	
Median (IQR), mean	0 (0–1), 0.81	0 (0–1), 0.78	>0.99

* Mann–Whitney test comparing *N* of PCI item distributions between the two IMD groups

**Table 4:** Concerns raised by 20 % or more of patients on the PCI, by IMD 2010 deprivation group

IMD Q1 (*n* = 47)		IMD Q2–Q4 (*n* = 40)	
Concern	%	Concern	%
Fear of the cancer coming back	43	Chewing/eating	45
Sore mouth^a^	43	Fear of the cancer coming back	43
Dry mouth^a^	29	Dental health/teeth	38
Dental health/teeth	28	Mouth opening	23
Chewing/eating	26	Pain in head and neck	23
Fatigue/tiredness	26	Dry mouth^b^	22
Pain in head and neck	21	Pain elsewhere	20
Sleeping	21	Swallowing	20
Speech	21		
Swallowing	21		

Some items were added later to the PCI:

^a^Based on *n* = 14

^b^Based on *n* = 9

**Table 5:** Members of staff that at least 10 % of patients would want most to see or be referred on to, by IMD 2010 deprivation group

IMD Q1 (*n* = 47)		IMD Q2–Q4 (*n* = 40)	
Member	%	Member	%
Dentist	26	Dentist	15
Surgeon	13	Surgeon	15
		Speech and language therapist	15

In regard to the total number of times, patients used the PCI the median (IQR) were 3 (1–6) times for patients in the more deprived quartile and 4 (2–6) times in the less deprived group, *p* = 0.21 Mann–Whitney test. The amount of follow-up was similar for both IMD groups, with 24-month survival of 83 % (SE 6 %) in both groups, *p* = 0.28 log-rank test for comparison of survival curves.

In respect of quality of life status, as reflected through the UW-QOL, there were indications of more patients from the most deprived quartile having significant problems in regard to mood (*p* = 0.004) and recreation (0.02), and a non-significant trend (36 vs 18 %, *p* = 0.09) in stating their overall quality of life as being less than good (i.e. as fair, poor or very poor). For each of these three results when the data were stratified into four groups by treatment (surgery, surgery, and RT/CRT) and by overall clinical stages (1–2 and 3–4), the rates for having significant problems were consistently higher for those living in the most deprived quartile (results not shown). Otherwise, there were no notable differences in regard to IMD status (Table 6).

**Table 6:** Association of IMD 2010 deprivation group with UW-QOL dysfunction, UW-QOL subscale scores, and UW-QOL overall quality of life scale

	Most deprived national IMD quartile Q1 (*N* = 47)	Less deprived national IMD quartiles Q2–Q4 (*N* = 40)	*p* value*
UW-QOL physical function subscale			
% With dysfunction			
Appearance	11 % (5/47)	15 % (6/39)	0.54
Swallowing	28 % (13/47)	13 % (5/39)	0.12
Chewing	11 % (5/47)	10 % (4/39)	>0.99
Speech	11 % (5/47)	3 % (1/39)	0.22
Taste	9 % (4/47)	8 % (3/39)	>0.99
Saliva	16 % (7/45)	16 % (6/38)	>0.99
Physical function subscale score (0–100): median (IQR)	72 (58–86), *n* = 47	73 (60–88), *n* = 40	0.51
UW-QOL social–emotional function subscale			
% With dysfunction			
Pain	23 % (11/47)	28 % (11/39)	0.63
Activity	11 % (5/47)	18 % (7/39)	0.37
Recreation	26 % (12/47)	5 % (2/39)	0.02
Shoulder	15 % (7/47)	8 % (3/39)	0.34
Mood	30 % (14/47)	5 % (2/39)	0.004
Anxiety	15 % (7/47)	10 % (4/39)	0.75
Social–emotional function subscale score (0–100): median (IQR)	73 (53–88), *n* = 47	76 (62–87), *n* = 40	0.18
% With less than good overall QOL	35 % (16/46)	18 % (7/39)	0.09

* Fisher’s exact test, apart from Mann–Whitney test to compare subscale scores

## Discussion

Although the PCI has the potential to improve the therapeutic alliance between patients and the clinical team, this is the first study which has looked at differences in PCI responses between socioeconomic groups and how this relates to self-reported quality of life. In the present study, the sample was recruited using consecutive sampling methods, which produced a good rate of use of the PCI (85 %), suggesting that most patients find the PCI feasible. However, the cross-sectional nature of this study limits the inferences that we can draw from the data; a longitudinal study would contribute to our understanding of how PCI responses and quality of life might change differentially with repeated use of the PCI for patients across the socioeconomic gradient. In addition, only patients with oral cancer were recruited for this study; therefore, the findings may not be generalisable to patients with other head and neck cancer diagnoses; however, PCI responses tend to be similar across a range of head and neck cancers, particularly with regard to Fear of recurrence [18], and therefore, it is likely that the findings would generalise to other head and neck cancers. Only patients from the Merseyside region were recruited for this study; therefore, the findings may not be generalizable to other UK regions. IMD area based measures are derived from patient post codes and do provide a relative measure of deprivation at small area level; however, within each area, there will be individual variation and we accept that measures of individual income, education level, and occupation may have provided a more sensitive measures of SES. The least deprived IMD quartile comprised only 11 patients and was combined with the second and third quartiles when comparing it to the most deprived quartile. In a larger sample of patients, we would have analysed differently by quartile. The comparison, however, reflects the particular nature of area deprivation within the Merseyside region and focuses in on the most deprived group of patients.

As shown in Table 1, 85 % of 106 eligible patients completed the PCI, and 89 % of patients from the most deprived quartile completed the PCI. This indicates that a large proportion of patients were able to use the PCI in clinic, despite lower levels of educational attainment and less familiarity with computer technology [19]. Patients can decline to use the PCI as part of their consultation and patients can miss the opportunity for a variety of reasons, such as computer failure, staffing levels and business of the clinic.

Table 2 shows that just over half (54 %) of the participants were living in the most deprived quartile. This is in line with previous research by Conway et al. [2], which found that individuals from low socioeconomic backgrounds were more likely to develop head and neck cancer. This relationship was mediated by alcohol consumption levels and smoking rates, suggesting that low SES individuals may be more likely to engage in unhealthy behaviours which increase their risk of head and neck cancer.

In addition, Table 2 shows that two-thirds (68 %) of male participants were living in the most deprived quartile, in comparison with 35 % of females (*p* = 0.004). In addition, younger patients were more likely to live in more deprived areas. These finding serve to emphasise the general characteristics of oral cancer patients. It would be expected that allowing for stage of cancer and treatment, male patients and younger patients would tend to report worse health-related quality of life outcomes. These groups could potentially benefit substantially by interventions when the sociocultural theory of health behaviours is considered. Males are less likely to seek help for health problems or engage in healthy behaviours than females due to norms of masculinity in which it is less socially acceptable for men to admit to experiencing poor health and instead ‘put up with it’ [20].

The number of PCI items selected by patients at their first clinic did not significantly differ between those in the most deprived quartile and patients in other quartiles, as can be seen in Table 3. This is interesting, as it might have been expected that those patients from lower SES would choose a few items to discuss in their consultation. The items raised on the PCI were similar across the socioeconomic gradient. In fact, the findings reported in Table 4 show that one of the two most common concerns selected on the PCI across the socioeconomic gradient was fear of recurrence (43 %). This is in line with research finding that fear of recurrence seems to remain a significant concern in a number of patients across a number of years following treatment, and a significant predictor of this seems to be problems with anxiety or mood as measured by the UW-QOL questionnaire [21]. Rogers et al. [22] found that a fear of recurrence screening question could be added to the UW-QOL questionnaire, which may help to identify those affected by fear of recurrence more adversely in review consultations. This would allow such patients to be referred to services which may help to alleviate their fear of recurrence concerns.

There were significant differences with regard to some items of the UW-QOL questionnaire, as can be seen in Table 6. Patients from the most deprived quartile reported significantly more problems with recreation (*p* = 0.02) and mood (*p* = 0.004) than patients from other quartiles, and there was a non-significant trend for patients from the most deprived quartile to rate their quality of life as being less than good (36 vs 18 %, *p* = 0.09). This is in line with studies by Rylands et al. [4, 5] which found that SES was associated with recreation and mood problems on the UW-QOL, and suggests that patients from more deprived backgrounds tend to suffer worse health-related quality of life particularly with regard to socioemotional issues, than patients higher up the socioeconomic gradient. Perhaps, measures could be put in place to identify patients from the lowest social strata who are experiencing especially poor quality of life to provide special support for this group.

There are a number of implications for the findings presented here. The finding that a large proportion of patients agreed to and were able to complete the PCI indicates that it would be feasible to implement this into regular care at head and neck oncology review clinics. This would be a method of facilitating patient-centred care; however, to date, research has not addressed whether the PCI has an effect on patient quality of life, and if so, how it affects quality of life. Doctor–patient communication can differ by SES [23], which can lead to poor health outcomes [24]; could the PCI affect quality of life through improved doctor-patient communication? This could in turn affect illness representations, which could also impact health-related quality of life [25]. Future research should explore these possibilities. The finding that fear of recurrence was one of the most commonly reported concerns on the PCI across the socioeconomic gradient suggests that identification of and interventions to address fear of recurrence concerns could benefit a large proportion of patients, potentially leading to improvements in quality of life. The PCI could be a useful tool for identifying fear of recurrence concerns, particularly if a fear of recurrence screening question was added [22]; however, interventions may vary depending on the severity of fear of recurrence; some patients may only require reassurance from their consultant, whereas others may need to be signposted to specialist psychological services.

In conclusion, this study found no significant differences in the use of PCI across the socioeconomic gradient. It is a means to help patients express their individual concerns during their routine follow-up clinic. There was notably worse quality of life, mood, and recreation in patients from more deprived backgrounds, and further research is required to assess whether interventions targeted specifically at this group could improve their outcome.
